# Secondary Pulmonary Hypertension and Right-Sided Heart Failure at Presentation in Grave's Disease

**DOI:** 10.1155/2012/762023

**Published:** 2012-11-07

**Authors:** Swapnil Panjabrao Ganeshpure, Gaurang Nandkishor Vaidya, Vipul Gattani

**Affiliations:** Department of Medicine, Grant Medical College and Sir J.J. Group of Hospitals, Mumbai 400008, India

## Abstract

A young female presented with evidence of right-sided heart failure and was subsequently found to have significant pulmonary artery hypertension (PAH). Because of her normal left ventricular function and pulmonary capillary wedge pressure, the most probable site of etiology seemed to be the pulmonary vasculature. All the common possible secondary causes of PAH were ruled out, but during the investigations, she was found to have elevated thyroid function tests compatible with the diagnosis of Grave's disease. The treatment of Grave's disease, initially by medications and subsequently by radioiodine therapy, was associated with a significant reduction in the pulmonary artery systolic pressure. The purpose of this case report is to highlight one of the unusual and underdiagnosed presentations of Grave's disease.

## 1. Introduction

Hyperthyroidism has been known to cause a variety of cardiovascular manifestations. In recent times, there have been reports of secondary pulmonary hypertension in patients with hyperthyroidism, though in most cases, this association led only to a mild and transient elevation of mean pulmonary artery pressure. A case of previously asymptomatic Grave's disease having the signs and symptoms of right heart failure at presentation is a rarity, and the association could be easily missed. This case presentation also emphasizes the need to look out for secondary and treatable causes of pulmonary hypertension before diagnosing a case as primary pulmonary hypertension.

## 2. Case Presentation

A 30-year-old Hindu married female, resident of Mumbai, presented with a 2-month history of dyspnea on exertion (NYHA Class II) which had worsened to dyspnea at rest since two days and pedal edema which she noticed two days back. She also complained of orthopnea, paroxysmal nocturnal dyspnea, and palpitations. She was a known case of rheumatic heart disease with mild mitral regurgitation. She was not on any medications, and her family history was non-contributory.

At presentation, she had mild tachycardia (heart rate = 108/min), normal blood pressure (120/70 mm of Hg), raised jugular venous pressure (8 cms.), pedal edema, and a thyroid swelling. Examination of the chest revealed hyperdynamic apex beat palpable at 5th intercostal space, a diastolic shock, and a left parasternal heave. On auscultation, she had a loud *P*_2_.

Her chest roentgenogram revealed cardiomegaly and a prominent proximal pulmonary artery. ECG showed right axis deviation, “P pulmonale,” and an evidence of right ventricular hypertrophy. Results of 2D echocardiography included evidence of rheumatic heart disease with mild mitral regurgitation, severe pulmonary artery hypertension with an estimated pulmonary systolic pressure of 70 mm of Hg, normal left ventricular function with an ejection fraction of 60%, normal left atrial dimensions, and left atrial volume index. She underwent workup for pulmonary hypertension with high resolution computed tomography of chest which showed mild cardiomegaly without any evidence of parenchymal involvement. Swan-Ganz catheter measured the pulmonary capillary wedge pressure as 11 mm of Hg (normal < 15 mm of Hg). The picture was consistent with the diagnosis of pulmonary artery hypertension. Computed tomographic pulmonary angiography showed no evidence of pulmonary thromboembolism. Ultrasonography of the thyroid showed bulky thyroid with increased vascularity and altered echotexture. Radioiodine uptake scan showed diffusely increased uptake in the thyroid gland.

Relevant laboratory results included serum T3 concentration of 450.93 ng/dL (normal 70–204 ng/dL), T4 concentration of 40.6 *μ*g/dL (normal 3.2–12.6 *μ*g/dL), and TSH < 0.01 *μ*IU/mL. HIV screen was nonreactive. Auto-antibody screen revealed positive antimicrosomal and anti-thyroglobulin antibodies and negative antinuclear antibody test.

The differential diagnoses that we ruled out during her admission include: left sided heart failure, cardiac valvular disease, pulmonary thromboembolism, HIV infection, pulmonary fibrosis, and collagen vascular diseases.

Patient was initially started on furosemide with minimal benefit. After the diagnosis of Grave's disease became clear, she was started on beta-blockers and carbimazole. Patient was subsequently sent to Tata hospital for radioiodine therapy.

A followup after 2 months with repeat 2D echocardiography showed pulmonary artery systolic pressure of 45 mm of Hg (Table 1), a significant decrease from the previous value.

## 3. Discussion

Pulmonary arterial hypertension (PAH) is defined as a mean pulmonary artery pressure (mPAP) of >25 mm Hg at rest or >30 mm Hg after exercise. The etiology of PAH can be divided into primary and secondary causes. The secondary causes are frequently treatable and should always be ruled out first before making the diagnosis of primary pulmonary hypertension. The association between PAH and hyperthyroidism was first reported in an autopsy case in 1980 [1].

The diagnostic dilemma in our case was the presence of a history of rheumatic heart disease but with normal left ventricular function. Though hyperthyroidism could have acutely complicated the previously present mitral regurgitation leading to decompensated left sided heart failure [2], the investigations failed to reveal so. Direct association between hyperthyroidism and severe pulmonary hypertension, though rare, has been reported before. In our case, this seemed to have led to the presentation of right sided heart failure.

Haran et al. [3] reported a case of a 33-year-old Asian man with 2 months of symptomatic Grave's disease, echocardiographic evidence of elevated right ventricular systolic pressure, and normal cardiac valves. This patient was treated with medications only- propranolol, propylthiouracil, steroids, and nifedipine, and repeat echocardiography 6 months later showed significant fall in right ventricular systolic pressure. 

Virani et al. [4] reported two similar cases of pulmonary hypertension with Grave's disease that underwent radioiodine therapy. They found that the treatment of hyperthyroidism resulted in an immediate reversal of the hemodynamic parameters and prompt symptomatic relief.

Suk et al. [5] performed serial echocardiographic examinations in 64 untreated patients with Grave's disease. The study found that the prevalence of PAH amongst the patients in the study was 44%. Follow-up echocardiography performed in the patients with PAH after treatment with antithyroid drugs, revealed that PAH had vanished in all except one patient. 

Marvisi et al. [6] studied 114 patients with hyperthyroidism (of which 47 had Grave's disease and 67 had nodular goiter) along with a matched control group. Mild pulmonary hypertension was found in 50 cases from the patient group which was again divided into 2 subgroups: those treated with methimazole and those with partial thyroidectomy. After a 120 day followup, the study concluded that the association between hyperthyroidism and mild and transient PAH is frequent and that methimazole causes a faster fall in mPAP compared to partial thyroidectomy.

Though the exact pathogenesis of this condition is not known, the mechanisms that have been debated in the literature include: increased pulmonary blood flow [5] or autoimmune process associated with endothelial damage [4]. Other possible explanations include increased cardiac output in hyperthyroidism or increased breakdown of intrinsic pulmonary vasodilators [7].

In summary, it can be commented that hyperthyroidism as a cause of reversible pulmonary hypertension has been reported previously. A few case series in the past have shown a frequent association between the two conditions, but most of the reported patients had mild and asymptomatic pulmonary hypertension. Such patients usually go undetected in clinical practice and the actual incidence of mild PAH in Grave's disease may in fact be greater than once thought. Exacerbation of PAH occurs in a fraction of these patients, but with the limited knowledge about the pathogenesis of this manifestation, it is difficult to predict the possibility of exacerbation in each of the previously asymptomatic patients. The prognosis in such cases depends solely on early detection and successful treatment of hyperthyroidism. The need for more research to better understand the mechanism underlying this association could not be over-emphasized. Better screening techniques that can predict the occurrence of severe pulmonary hypertension in patients with hyperthyroidism can save the patient from a potentially fatal complication.

Moreover, in rare circumstances, severe pulmonary hypertension may be the sole manifestation of hyperthyroidism. In such cases, this association could easily be missed, resulting in wrong diagnosis and treatment of a potentially reversible condition. With our case presentation, we try to highlight one such case of severe exacerbation of pulmonary hypertension and right side heart failure which on further workup was found to have Grave's disease. We would, therefore, like to emphasize the need for a complete workup of patients with pulmonary hypertension to rule out secondary causes of PAH. Primary PAH carries a poor prognosis and should always be a diagnosis of exclusion.

## 4. Conclusion

From the case and the review of literature, we can conclude the following.

- In patients with pulmonary hypertension not related to left heart disease, a search must be made for other reversible causes before making the diagnosis of primary pulmonary hypertension.
- Hyperthyroidism is quite frequently associated with mild and transient pulmonary hypertension, than previously thought and is usually reversible with treatment.
- In rare circumstances, pulmonary hypertension secondary to hyperthyroidism can be severe enough to present with right heart failure and should be included in the differential diagnosis when other common causes have been ruled out.
- More research is needed to better understand the pathogenesis of this association and the role of thyroid hormones and autoimmune factors in its causation.

## Figures and Tables

**Table 1:** Comparison of the patient's findings before and after treatment with radioiodine.

	Before treatment	After treatment
Serum TSH	<0.01 *μ*IU/mL	0.6 *μ*IU/mL
Serum T4	40.6 *μ*g/dL	12.0 *μ*g/dL
Pulmonary artery systolic pressure	70 mm of Hg	45 mm of Hg
